# From concept to approval: human genomic data integration with population observational data – insights from a Belgian pilot study

**DOI:** 10.1186/s13690-025-01581-9

**Published:** 2025-04-24

**Authors:** Nina Van Goethem, Johan Van der Heyden, Pierre Smith, Wannes Van Hoof, Harlinde De Schutter, Melissa Van Bossuyt, Karin De Ridder, Marc Van Den Bulcke, Emilie Cauët

**Affiliations:** 1 Present Address: Scientific Directorate of Epidemiology and public health, Sciensano, J. Wytsmanstraat 14, Brussels, 1050 Belgium; 2 Institute for Health and Society (IRSS), Université catholique de Louvain, Brussels, Belgium; 3 Belgian Cancer Registry, Brussels, Belgium; 4 Data Protection and Information Security, Sciensano, J. Wytsmanstraat 14, Brussels, 1050 Belgium

**Keywords:** Public health genomics, Precision public health, Real-world data, Whole-genome sequencing, Data linkages, Human genomic data, Population observational data

## Abstract

Linking genomic data with population-level observational data sources offers a powerful approach to advance public health genomics research, providing a more comprehensive view of health outcomes by incorporating information on various health determinants. However, integrating data from scattered sources poses significant challenges. Genomic data, with its unique identifying properties and ethical concerns, is particularly sensitive and requires strict security measures and transparent participant communication to ensure confidentiality and maintain public trust. In Belgium, a pilot study has been set up to evaluate genetic and non-genetic health determinants associated with cancer. As a crucial step towards this scientific objective, the study also aims to assess the feasibility and complexity of linking genomics data with national population-based datasets. The process, ranging from conceptualisation and data discovery to securing approvals and finalising agreements, took two years. Each phase of this process offers opportunities to improve efficiency, enhance coordination between stakeholders, and address legal and ethical challenges. Establishing comprehensive, interoperable data catalogues can facilitate data discovery, while standardising data access requests can simplify processes. Pre-established partnerships or agreements can reduce administrative burdens and consequently, improve the timeliness of the research. Additionally, planning for sustainability in advance and rethinking consent procedures could reduce ad hoc approval procedures and support structural solutions for secondary use and linkage of health data in the public interest. This paper highlights practical challenges and considerations relevant to data linkage studies in general, offering insights for researchers conducting integrated public health genomics research.

**Supplementary Information:**

The online version contains supplementary material available at 10.1186/s13690-025-01581-9.

**Table:** 

Text box 1. Contributions to the literature
• Blood samples from health survey participants, representative of a target population, provide a strong foundation for public health genomics research. Subsequent linkages with existing population-level observational data sources enables to study multiple health determinants simultaneously.
• Many barriers to data linkages stem from unclear and varying interpretations of ethical and legal frameworks, leading to significant time spent understanding the scope of existing approvals, and related to that, the required additional procedures.
• Variability in procedures across institutions complicates data access, underscoring the need for standardised processes. A unified national level (and additionally, cross-border) legal framework with aligned regulations and common standards would support (genomic) data reuse.

## Background

Public health genomics refers to the integration of genomic information into public health practice with the aim of improving the health of populations [1]. The advances and increased use of genomic technologies have supported the inception of precision public health, aiming to provide the right intervention to the right population at the right time [2]. However, it is essential to assess how genomics integrates into a comprehensive health model that considers a combination of health determinants. The health of populations and individuals is determined by the dynamic interplay between genetic factors, socioeconomic conditions, lifestyle, environmental exposures, and the quality and accessibility of medical care.

Complex diseases, like cancer, highlight the need for this integrated approach due to their multifactorial nature. Genomic alterations, environmental exposures, lifestyle, and social determinants all contribute to cancer risk and progression, along with early detection. Therefore, understanding complex gene-environment interactions [3] and effectively stratifying populations into different risk groups based on their overall disease predisposition requires combining information on individual variability in genes, environment, lifestyle, and social factors. To achieve this, it is often required to incorporate data from various sources, as the necessary information is traditionally not collected in a single data source [4].

Combining routinely collected observational data sources, such as electronic health records, disease registries, survey-based population studies, medical and pharmacy claims, laboratory data, and administrative records, offers a wealth of information while reducing the need for new data collections. Data initially gathered for specific purposes (i.e., primary use) like patient diagnosis, follow-up or administration, are also referred to as real-world data (RWD) [5, 6]. These data can later be repurposed for secondary uses, including research, innovation, and policy-making. The European Health Data Space (EHDS) aims to facilitate the primary and secondary use of health data across borders, by providing common rules, standards and infrastructures, and a governance framework. In March 2025, the EHDS regulation entered into force, marking the beginning of the transition phase towards a gradual implementation.

Currently, regulations concerning genomic data are still evolving with ongoing developments in the EHDS implementation. Under the General Data Protection Regulation (GDPR), genomic data is considered to be particularly sensitive because of its unique identifying properties, predictive health information, privacy risks, familial implications, security challenges, and ethical concerns [7, 8]. Ensuring the confidentiality and security of genomic data involves robust encryption methods, secure data storage solutions, and strict access controls. Integrating genomic data with other diverse data sources further complicates privacy considerations in public health genomics research, requiring new legislative solutions [9]. Moreover, ethical considerations require transparency with participants regarding how their data will be used, potential risks, and the measures in place to protect their privacy. Addressing these concerns is essential to maintain public trust and enable the effective use of integrated data in public health genomics [8].

Subsequently linking existing observational data sources to genomics data is not only a cost-effective approach, but often the only feasible way to obtain the required comprehensive data for public health genomics research. Horizontal data linkage refers to connecting and integrating information from multiple datasets or sources that relate to the same individual, family, place, or event to provide a more comprehensive view [10]. However, horizontally linking data from scattered sources, such as those held by different partners or institutions, presents several challenges due to variations in data access procedures, data standards, and the application of privacy regulations.

The objective of this manuscript is to describe the experiences and insights gained from the initial phases, spanning concept development and data discovery to the design of the data flow architecture and authorisation, of a pilot study linking genomics data with existing national population-based datasets in Belgium. While the pilot study itself addresses specific research questions related to the relative importance and the interaction between genetic and non-genetic health determinants for the risk of cancer, and potentially other chronic diseases, this manuscript focuses on the challenges and complexities encountered throughout this process, from concept to approval. It aims to assess the technical, operational, temporal, ethical and legal feasibility of such data linkages in the field of public health genomics. The lessons learned from this experience can be leveraged to enhance future data collections and linkages, and ultimately to build a stable infrastructure that shifts from ad hoc, project-based linkages to a more systematic approach. Although specific to the Belgian context, the findings offer valuable guidance for researchers aiming to undertake integrated public health genomics research in other settings.

## Implementation of a data linkage pilot study in the field of public health genomics in Belgium

### Objectives of the pilot study

A pilot study has been set up at the Belgian national level to link genomic data with other population-level datasets, with the prospect of answering research questions on the role of genetics in cancer disease risk and how genes interact with other health determinants. Before addressing these scientific questions, the study first aims to assess the feasibility and complexity of such data linkages, thereby addressing the technical, organisational, temporal, and operational challenges, alongside the ethical and legal considerations related to data governance, to enhance future public health genomics efforts.

### Steps of implementing a data linkage study

**Fig. 1:**
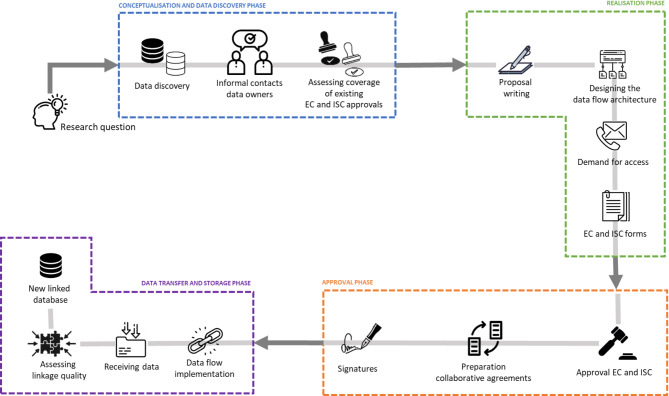
The consecutive steps of a data user’s journey to implement a data linkage study. EC: Ethics Committee; ISC: Information Security Committee

The data user journey to link human genomic data with relevant population-based observational data involves several key phases as depicted in Fig. 1. The conceptualisation and data discovery phase (Fig. 1, blue box) begins with formulating the research question and the identification of relevant datasets. This is followed by identifying the appropriate contacts, informal engagement with data holders, and a review of existing legal and ethical frameworks. Next, the realisation phase (Fig. 1, green box) involves developing a comprehensive study protocol and supporting documents, designing secure data flow procedures, submitting data requests, and preparing application forms for the Ethics Committee (EC) and the Information Security Committee (ISC). The approval phase (Fig. 1, orange box) then follows, during which formal approvals are obtained, and collaborative agreements have to be signed with all partners. Finally, the data transfer and storage phase (Fig. 1, purple box), which is beyond the scope of this paper, involves the actual implementation of the data linkages, conducting a small-cell risk analysis (SCRA), securely transferring the data and integrating it to create a new linked database.

### Description of the study population and leveraged data sources

The pilot study’s conceptualisation and data discovery phase was initiated by exploring existing data sources and opportunities for obtaining genome sequencing data from a representative population sample. Blood samples available for sequencing collected within an established population-based health survey framework offered an excellent foundation for conducting research in public health genomics.

**Fig. 2:**
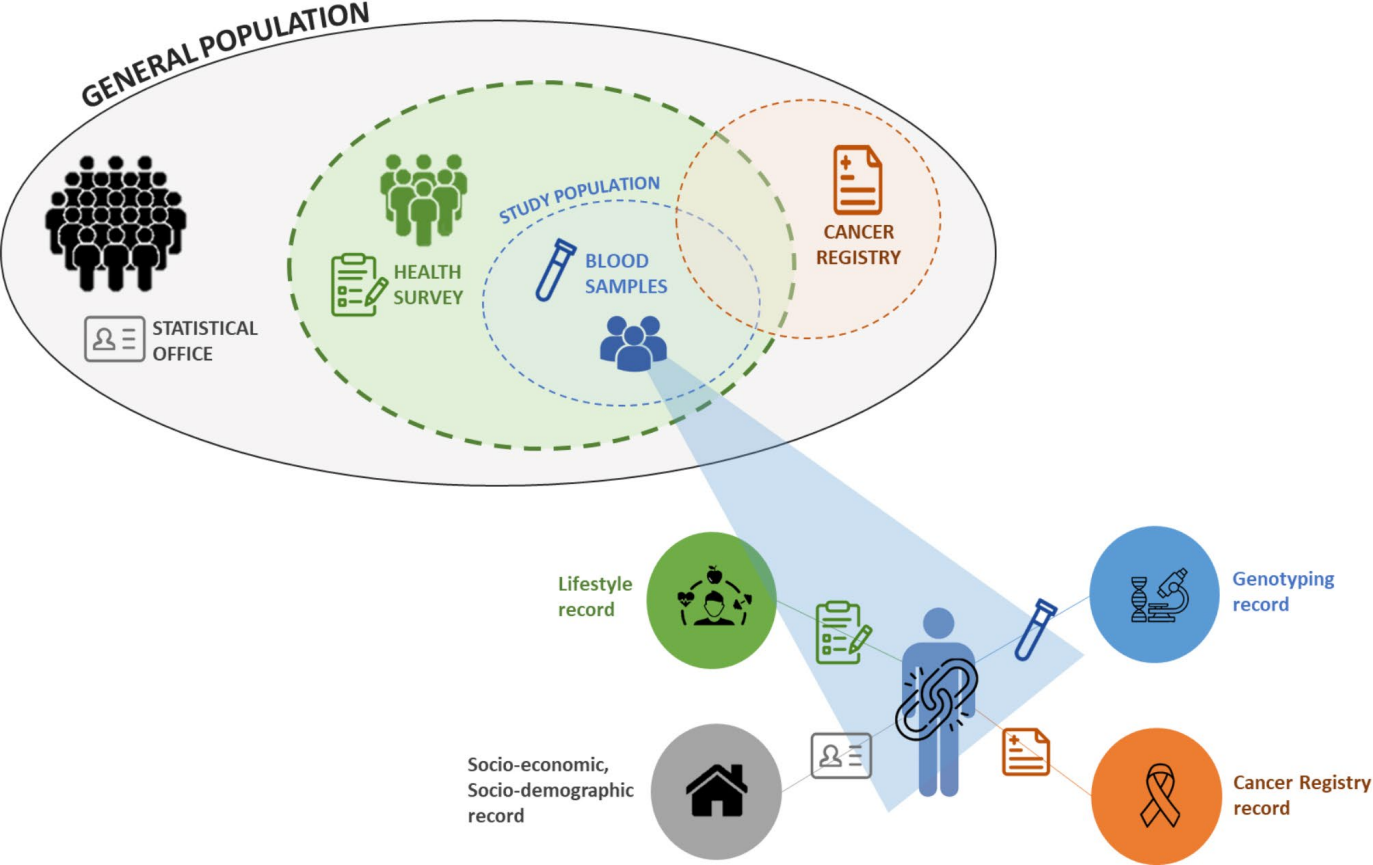
Study population and linked data sources in a pilot study linking human genomic data to relevant population-based observational data at the national level in Belgium. The diagram illustrates the relationship between the general population (grey), health survey participants (green), the health survey participants with an available blood sample and who gave consent for subsequent DNA analysis (blue), and cancer patients (orange). The study population (blue) is a representative sample drawn from the general population. For each individual in the study population, detailed information is available at the time of the survey year (i.e., 2018), including lifestyle factors collected from the health survey (Belgian Health Interview Survey, BELHIS), socio-economic and demographic characteristics obtained through the National Statistical Office (Statbel), genotyping results obtained through whole-genome sequencing of collected blood samples, and any eventual cancer diagnosis recorded in the Belgian Cancer Registry (BCR) during the period 2004–2022

The study population encompasses health survey participants from the Belgian Health Interview Survey (BELHIS) [11] for whom blood samples have already been collected through the Belgian Health Examination Survey (BELHES) and are available for DNA sequencing (Fig. 2). Additionally, a linkage with several population-based data sources was envisioned. The availability of a unique identifier enabling linkage with other population datasets allowed for the integration of genomic data with a wide range of health and demographic information. This resource-efficient approach leverages existing survey and administrative data, reducing the need for new data collection efforts. Following the respective linkages between different data sources, detailed individual-level information will be available for each participant, including the genotyping results, lifestyle factors collected from health surveys (BELHIS and BELHES), socio-economic and demographic characteristics obtained from the National Statistical Office (Statistics Belgium, Statbel), and any cancer diagnoses recorded by the Belgian Cancer Registry (BCR).

### Health survey data

The Belgian Health Interview Survey (BELHIS) is a national, cross-sectional household survey conducted every five years since 1997 by Sciensano, the Belgian Institute for Health. The target population of the BELHIS consists of all persons with residence in Belgium, including the institutionalised elderly, with no restrictions on age or nationality. Participants are selected through a multistage, stratified-sampling design from the national population register. By applying population weights to adjust for oversampling of certain groups, representativeness is approximated as closely as possible. It is regarded as the principal reference in terms of population-based health survey data in Belgium and holds a vast amount of information on health status (physical and mental health), health-related attitudes and behaviours (lifestyle), use of health care facilities and preventive services (e.g., cancer screening), and perception of the physical and social environment. The survey methodology is described elsewhere [11]. A key purpose of the BELHIS is to collect data at the level of the total population, including people who do not make use of health services [12].

In 2018, the Belgian Health Examination Survey (BELHES) was organised as a second stage of the BELHIS for a subset of the participants to obtain objective measurements through clinical examinations and analysis of biological samples. The sampling frame of the BELHES consisted of all persons who participated in the BELHIS 2018 except for minors (< 18 years), BELHIS participants for whom a proxy interview was conducted and residents of the German Community, which is a minority in Belgium (< 1% of total population). Details on the sampling scheme of the BELHES can be found elsewhere [13].

Out of 11,611 BELHIS participants in 2018, 4,918 were eligible to be invited to the BELHES. Among these invited participants, 1,184 individuals eventually took part in the BELHES. The study population of the current pilot study includes the BELHES participants who consented to provide a blood sample for DNA analysis and whose blood samples are available in Sciensano’s biobank. This study population comprises 981 individuals.

As a first step, the codebooks of the BELHIS and BELHES datasets were consulted to identify relevant variables for the proposed research questions, and a detailed data request was submitted to Sciensano’s Health Survey team.

### Human genomic data

Genomic data is obtained through Whole-Genome Sequencing (WGS) for the BELHES participants for whom a blood sample is available in Sciensano’s biobank and who signed an informed consent for DNA analysis in 2018 (*n* = 981). For a first subset of samples (*n* = 100), WGS data has already been obtained in 2019 within the scope and timeframe of the BELHES study period (Phase I) and these data will be retrospectively reused within the pilot study. The generated DNA sequencing data are stored as FASTQ files on a secured internal network server at Sciensano. After processing the raw genomic data, a VCF file is created containing both Single Nucleotide Polymorphisms (SNPs) and Insertions and Deletions (INDELs). Sequencing data for the remaining residual blood samples in Sciensano’s biobank will be obtained through prospective analyses in future phases, contingent on funding opportunities.

### Cancer registry data

The Belgian Cancer Registry (BCR), founded in 2005, is a population-based registry with national coverage from the incidence year 2004 onward, collecting information about all new cancer diagnoses in Belgium and their follow-up. Its activities are legally grounded in coordinated law concerning the execution of healthcare professions of 10 May 2015. The law mandates that hospitals with oncological care programs and pathology, clinical biology and haematology laboratories must register all new cancer diagnoses. For oncological care programs, collected information is based on the standard cancer registration form. For laboratories, BCR receives a limited set of structured data accompanied by the full text reports. Additionally, it authorises the use of the national security number (used for social security purposes and identical to the national register number for Belgian residents) as the patient’s unique identifier and permits linkage with other administrative databases to facilitate activities like active follow-up on vital status. For the pilot study, data on tumour type (using ICD-10 codes), stage at diagnosis, and date of diagnosis (limited to the quarter of the year) were selected from the BCR. These data covered incidence years from 2004 to 2022, the range available at the time of the request.

### Administrative and socioeconomic data

The National Statistical Office (Statbel) collects, produces and disseminates relevant figures on the Belgian economy, society and territory. The data collection is based on administrative data sources, i.e., existing data from public or private institutions, and surveys conducted among citizens and enterprises. Key administrative data sources include the National Register of Natural Persons (RNPP), the census, and tax information from the federal department of Finance. For individuals in the pilot study, microdata have been requested regarding their status in the National Register, and, if deceased, the underlying cause of death. Further, nationality, country of birth, and origin were requested as proxies for genetic background to account for population stratification in the genetic analysis. Additionally, sociodemographic and socioeconomic information, including civil status, household status, educational level, employment status, and net taxable household income decile, have been collected for the study population. For variables that could change over time, the data have been requested for incidence years from 2018 until the most recent year available at time of data delivery. The microdata request form was prepared in collaboration with an assigned statistician.

### Designing the data flow architecture

Integrating data from multiple, scattered sources presents several challenges, particularly when it comes to accurately linking data while ensuring privacy protection. The use of unique identifiers supports a deterministic linkage approach, ensuring that data from various sources can be reliably and accurately linked.

In Belgium, each resident is assigned a National Register Number (NRN) at birth or upon registration, which serves as a unique identifier. The NRN is used across various domains, including healthcare, taxation, social security, and employment, enabling efficient interaction between citizens and public services. In this study, the NRN will be used to link selected variables from the BELHIS, BELHES, BCR and Statbel datasets at the individual level.

To safeguard privacy and prevent unauthorised access, it is essential to apply the separation principle, i.e., the process of linking datasets together has been separated from the actual process of extracting data for analysis [14]. Consequently, none of the parties involved in the linkage procedure have access to both linked sensitive data and NRNs. This is where a Trusted Third Party (TTP) plays a crucial role, ensuring a secure and independent linkage process. Given the complexity of this process, multiple coding procedures are employed to protect privacy and ensure data security.

For the pilot study, eHealth was consulted as the TTP to design the data flow architecture. They provide a range of essential services that facilitate secure and efficient data management and exchange, including user and access management, end-to-end encryption through the eHealthbox, secure electronic mailboxes, pseudonymisation and anonymisation, and timestamping, among others.

**Fig. 3:**
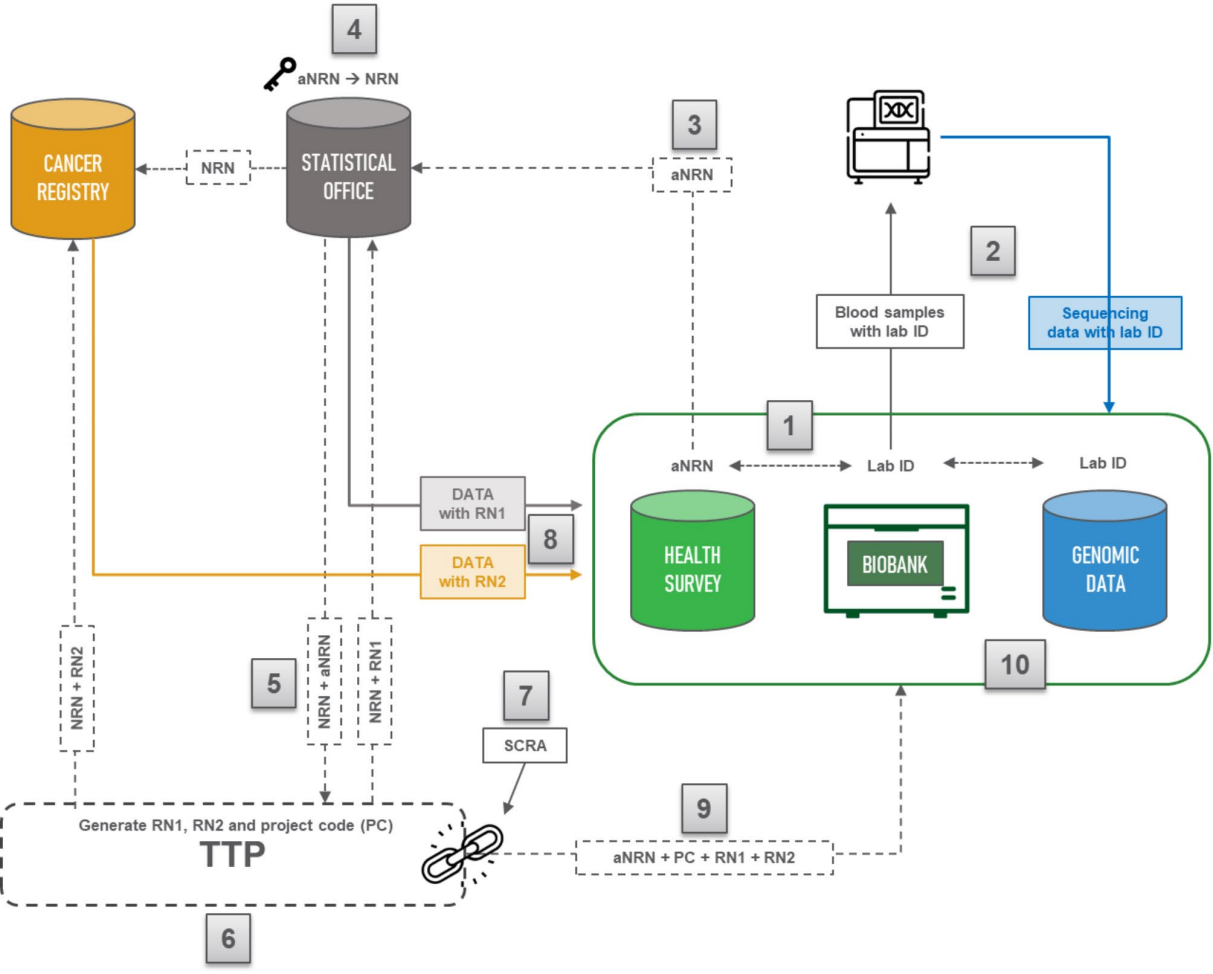
Step-by-step overview of the data flow to enable data linkage between health survey data, genomic data, administrative data and cancer registry data in a pilot study conducted at the national level in Belgium NRN: national register number; aNRN: pseudonymised national register number; RN: random number; TTP: trusted third party; PC: project code; SCRA: small cell risk analysis. The figure shows the design of the data flow, where data (represented by solid arrows) and personal identifiers (represented by dashed arrows) are consistently kept separate

Figure 3 illustrates the data flow and the roles of each partner involved. Statbel holds the key to obtain the NRN based on the code assigned to participants in the health survey, which is a pseudonymised NRN (aNRN). Statbel selects the NRNs of the pilot study participants and transmits this selection to the BCR and the TTP.

Upon receiving the list of NRNs and corresponding aNRN, the TTP generates a project code (PC). To maintain privacy, the PC is not sent together with the NRNs; instead, the TTP generates a random number (RN1) for Statbel and a second random number (RN2) for the BCR. Both the BCR and Statbel then send the retrieved data along with the corresponding RNs to Sciensano.

Finally, authorised researchers receive the link between the aNRN, RN1, RN2, and PC from the TTP, enabling them to link the datasets while retaining only the PC. A SCRA, serving as an additional risk analysis and a standard ISC requirement, will be conducted by an independent organisation designated to do so. Ultimately, only pseudonymised data will be available to authorised researchers on a secure server.

1. Within the Belgian public health institute – Sciensano (green), the health survey data and blood samples with their lab ID are linked through one common pseudonymised project number (aNRN). Selected variables are extracted from the health survey database.
2. The blood samples, stored in Sciensano’s biobank together with their lab ID, have been sent for sequencing. The generated sequencing data are stored at Sciensano.
3. Sciensano submits the aNRN of the study population to the national Statistical Office (Statbel).
4. Statbel has the key the obtain the NRN based on the aNRN and subsequently submits the list of NRNs to the Belgian Cancer Registry (BCR) in order to retrieve the selected variables for these individuals. Similarly, Statbel retrieves the requested microdata based on the NRN.
5. Statbel sends the list of NRNs and corresponding aNRNs to the TTP.
6. Upon receiving the list of NRNs and corresponding aNRN from Statbel, the TTP will generate a PC. As the PC cannot be send together with the RRN, the TTP also generates a RN1 and RN2 to send to Statbel and the BCR, respectively.
7. A SCRA is carried out.
8. Both the BCR and Statbel will send the retrieved data accompanied by the corresponding RNs to Sciensano.
9. The authorised researcher(s) receive the link between the aNRN, RN1, RN2 and PC from the TTP.
10. Authorised researchers can link the respective datasets and only keep the PC.

### Data access procedures

After identifying the relevant data sources and designing the data flow, the process continued by assessing the coverage of existing approvals and defining the scope of the request(s) specific to the pilot study. The advice of the respective committees, along with early consultations with Sciensano’s Data Protection Officer (DPO) and legal office, was crucial in outlining the study’s compliance strategy. Based on their guidance and in close collaboration with the different data providers, the study protocol was drafted, along with other relevant documents such as a Data Management Plan (DMP) and a Data Protection Impact Assessment (DPIA).

### Ethics committee

Ethical conduct is a fundamental concern in any research involving humans. This means protecting participants not only from risks to their physical and mental health but also from risks to their privacy. Given the physical nature of taking blood samples, all participants included in the pilot study have signed an informed consent form (ICF) before their participation in the BELHES 2018. According to this ICF, any future studies using DNA analysis results from participants who agreed to such use will only be conducted with approval from an ethics committee. Further clarifications on the coverage of the existing ethical approval and ICF was sought from the respective ethics committee.

**Fig. 4:**
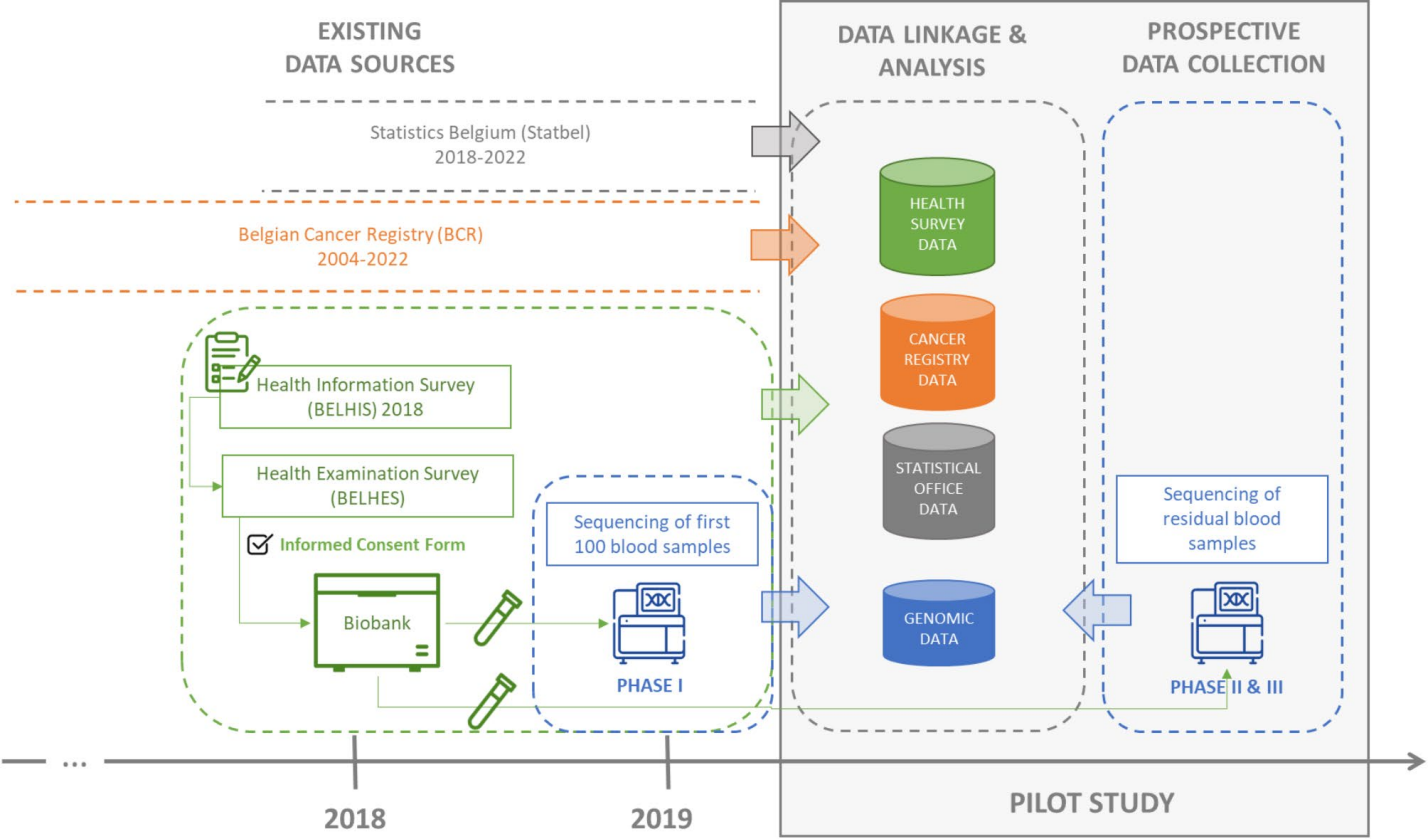
Scope of the ethics committee demand for the pilot study linking human genomic data to relevant population-based observational data conducted at the national level in Belgium BELHIS: Belgian health interview survey; BELHES: Belgian health examination survey

Finally, the scope of the ethics demand for the current pilot study (see Fig. 4) covered both the prospective collection of new DNA sequencing results and the analysis of the linked study database, which includes existing data collections that have been leveraged and included in the pilot study. The DNA sequencing data from the initial 100 samples, obtained within the scope and timeframe of the BELHES study, were included alongside existing data from BELHIS, BCR, and Statbel.

The pilot study was submitted to the Medical Ethics Committee as a ‘prospective study with human body material’, given the planned DNA sequencing of residual blood samples stored in Sciensano’s biobank. The study protocol, along with the DMP and other supporting information, was provided through the EC’s online portal.

### Information security committee

The Belgian Information Security Committee (ISC), established in 2018, consists of two Chambers: one for Social Security and Health, and another for Federal Government matters. It is an independent neutral body appointed by the Parliament to assess GDPR compliance for (further) processing of personal data related to health such as data linkages. Before a positive deliberation, the ISC ensures that the request complies with GDPR legislation. The ISC also establishes the information security conditions to be followed in data exchange and linkages. However, it is important to note that the ISC is a coordinating body addressing information security issues and does not function as a supervisory authority enforcing the GDPR in Belgium, which is the responsibility of the Data Protection Authority (DPA).

Approval for both the BELHIS and the BELHES 2018 was granted by the Sectoral Committee Social Security and Health (the precursor of the ISC) before their initiation. Given the planned data linkages in the pilot study, which involve combining data from BELHIS and BELHES 2018 with data from BCR and Statbel, approval from the ISC was required to ensure compliance with privacy and data protection regulations.

Under the GDPR, individual privacy protection is balanced with other important interests like public interest, national security, and law enforcement. Article 5 of the GDPR outlines fundamental principles that must guide personal data processing, including lawfulness, proportionality, accuracy, data minimisation, storage limitation, and integrity and confidentiality. The principle of proportionality requires researchers to only collect and process the necessary data, using it only for the intended purpose, and implementing appropriate safeguards to protect individuals’ privacy rights. This principle aligns with the concept of data minimisation, ensuring that only personal data which is adequate and relevant for the purposes of the processing is collected and processed. Consequently, only selected variables relevant to our research questions from the BELHIS, BELHES, BCR and Statbel databases have been requested for the specific objectives of the pilot study.

To preserve privacy and prevent the disclosure of sensitive information, the linkage process (outlined in Fig. 3) followed the separation principle of linkage and analysis processes. This means that the linkage has been outsourced to another organisation, specifically a TTP, which has access to a set of identifiers. Meanwhile, the researchers analysing the linked data only have access to de-identified pseudonymised data.

This information has been provided in the ISC demand template and related documents, including an DMP, a Processing Activity Assessment, and a DPIA, following the support of the DPOs of Sciensano, BCR and Statbel.

### Collaborative agreements

Formal agreements that regulate interactions, collaborations, data sharing, and confidentiality between different organisations or external partners ensure that all parties are aligned on their obligations and protections. These legal documents define the roles, responsibilities, timelines, and expectations of each party involved, outline procedures for data transfer, and establish terms for protecting sensitive and proprietary information.

​The data from BELHIS 2018, which also includes the data from previous editions, is subject to the GDPR, the Framework Act of July 30, 2018, and the Act of September 5, 2018 establishing the ISC. For requests involving only variables listed in the anonymous HIS 2018 database codebook, a streamlined data transfer procedure is used, requiring only a Data Transfer Agreement (DTA) to be signed. However, ISC approval is necessary for non-anonymous data.

Similarly, the BCR adheres to strict norms and confidentiality procedures when sharing its sensitive data, whether with the public, authorities, researchers, students, the media, or pharmaceutical companies. Upon ISC approval, pseudonymised data can be released following the signing of a DTA.

The sharing of Statbel’s microdata is also regulated under the GDPR (Regulation No 2016/679) and the Framework Act of July 30, 2018, on the protection of natural persons regarding the processing of personal data. Statbel further adheres to conditions set by the Statistical Supervisory Committee, the ISC, and agreements with relevant data providers. To initiate a data request for the pilot study, a formal application was completed using a standardised form available on Statbel’s website, with each requested variable justified for its relevance to the research. The application was reviewed by a multidisciplinary committee within Statbel, with input from Statbel’s DPO. Once approved, a confidentiality contract was drafted and signed by Sciensano’s legal representative. The requested data can be released after the signed contract is received and a positive ISC deliberation is granted.

Before the data could be exchanged, a TTP Global Document was prepared by the eHealth Platform’s TTP Service team. This document summarises the data exchange procedure, including details on the senders, recipients, and transmission process, in accordance with the deliberations of the ISC. It was then required to be signed electronically by all parties involved in the pilot project.

## Challenges encountered during the implementation of a data linkage pilot study in the field of public health genomics in Belgium

### Prolonged timeline and delays in study approval

The pilot study’s conceptualisation and data discovery phase was initiated in January 2023, initially focusing on Sciensano’s health survey and genomic data without considering data linkages. The first submission to the EC was made in March 2023, with feedback received in April 2023. Parallel exploratory meetings with the BCR, Statbel, and TTP explored the feasibility of linking cancer registry and socioeconomic data to Sciensano’s health survey and genomic data. This, combined with EC feedback, led to a reassessment of the study’s scope, requiring revisions and a resubmission in December 2023. As a result, a significant portion of the timeline was dedicated to identifying relevant data sources and variables, engaging in informal contacts with various data providers, and determining what was feasible and required in terms of ethical and legal obligations.

Further challenges arose in preparing the ISC request due to recursive feedback loops with data providers and the high workload of Sciensano’s DPO, causing significant delays. After securing agreements from all partners, the study was submitted to the ISC in January 2024. EC approval followed on March 19, 2024, and ISC approval on May 3, 2024. Following these formal approvals, the initiation of collaborative agreements began, but delays in coordinating these actions with multiple stakeholders persisted.

**Fig. 5:**
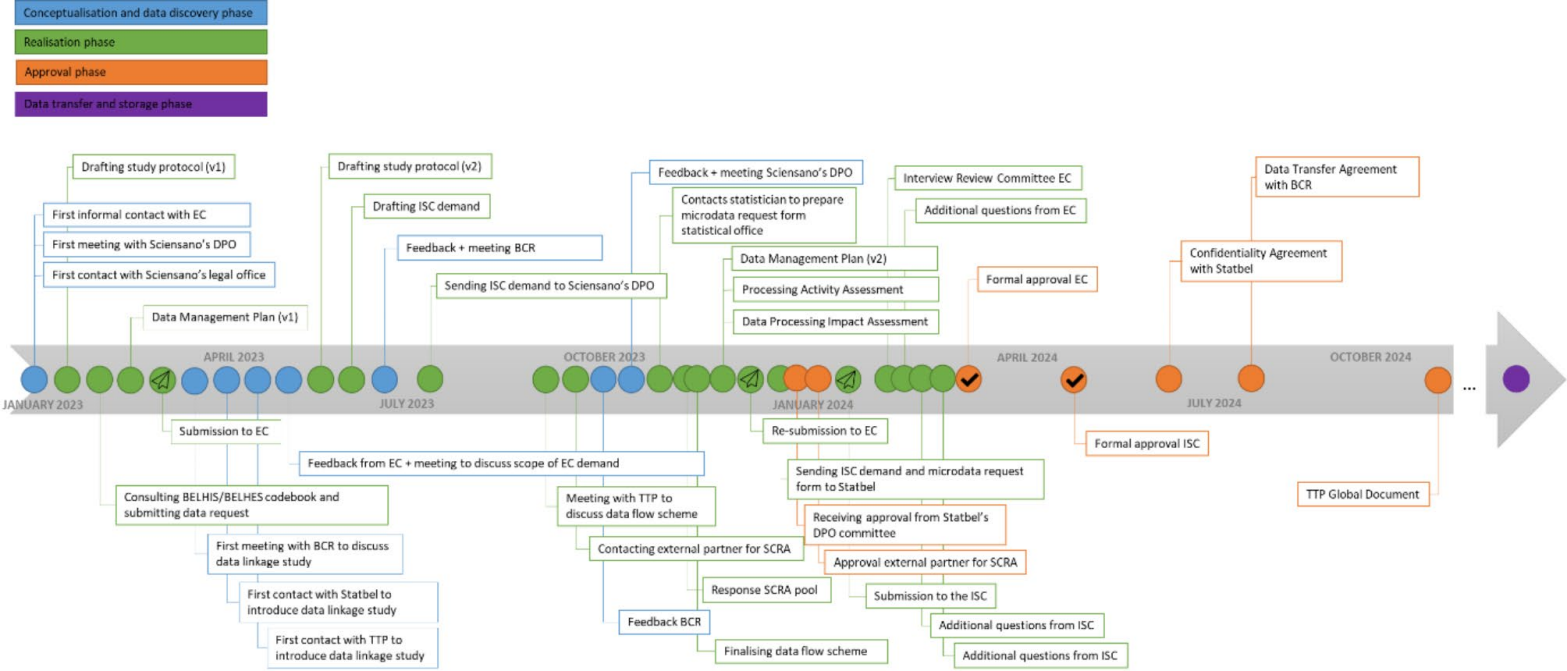
Timeline of implementing a pilot study linking human genomic data to relevant population-based observational data conducted at the national level in Belgium (January 2023 – January 2025). EC: Ethics Committee; TTP: Trusted Third Party; SCRA: Small Cell Risk Analysis; ISC: Information Security Committee; DPO: Data Protection Officer; BCR: Belgian Cancer Registry; Statbel: Statistics Belgium

A detailed overview of all steps and respective dates is provided in an additional table [see Additional file 1], as well as presented in Fig. 5. The entire process, from the conceptualisation and data discovery phase initiated in January 2023 to the finalisation of the approval phase in early January 2025, took two years to complete. The delays were largely due to navigating the data landscape, managing the administrative burden, coordinating with the various stakeholders involved in the data linkages, and complying with ethical and legal procedures. Furthermore, many steps had to be carried out sequentially, meaning the responsiveness and availability of key individuals had a considerable impact on the timeline.

### Clarifying ethical approvals, consents, and recontacting requirements

Interactions with the EC highlighted significant challenges in understanding what was covered by existing ethics approvals and the ICF, and in defining the scope of the ethics demand for the current pilot study. As stated in the original ICF that had been signed by the study participants in the context of the BELHES, future studies using these results require a separate ethics approval. However, determining the ICF’s coverage was complex due to its ambiguity, leading to different interpretations regarding what was permitted. This complexity was further compounded by the various phases of DNA sequencing acquisition over time, which is dependent on funding opportunities. As such, the pilot study foresees to use both existing sequencing data and prospective data collections (i.e., generating sequencing data in subsequent phases) (see Fig. 4).

The EC’s primary concerns included whether new informed consents would be requested from participants who agreed to be re-contacted and how the researchers would handle participants’ requests to communicate results to their doctors. These concerns were not applicable to the pilot study, as no additional data would need to be collected from participants. Further, the researchers had to clarify that the pilot study is considered as a proof-of-concept with low statistical power, aimed at assessing the feasibility of establishing the necessary infrastructure to connect genomic data with other relevant population-wide data and to subsequently associate cancer polygenic risk scores and phenotypic outcomes. Given the pilot study’s preliminary phase, it was concluded that the results would not be sufficiently robust to report back to participants.

### Navigating administrative and legal procedures

A significant amount of effort and time was spent determining the administrative steps necessary to initiate data access requests. Each data provider, representing different institutions, has its own procedures and internal workflows, complicating the process. Guidelines are frequently not publicly available, necessitating the reliance on informal contacts to gather practical information.

It was challenging to determine the proper sequence of steps, particularly when attempting to carry out actions in parallel to save time. Many actions required input from other steps, which ultimately delayed the overall process. In addition, the information required for the various templates, online portals, and forms was often very similar, resulting in repetitive tasks and the need to frequently cross-reference documents to ensure consistency and alignment.

## Recommendations and future perspectives for preparing data linkage studies in the field of public health genomics

Reflecting on the implementation of the current pilot study reveals several recommendations for data linkage studies. Early engagement with all stakeholders, including data providers, legal teams, and ethics committees, is crucial for understanding constraints and avoid unnecessary feedback rounds. Having legal expertise with a clear understanding of the project’s needs within the team could avoid delays from external consultations. Determining the sequence of steps early on and identifying those that can be completed in parallel helps to save time and improve efficiency. Having the different templates readily available ensures consistency and minimises redundant work, given that much of the required information overlaps across forms. Finally, setting realistic timelines helps to account for potential delays.

**Fig. 6:**
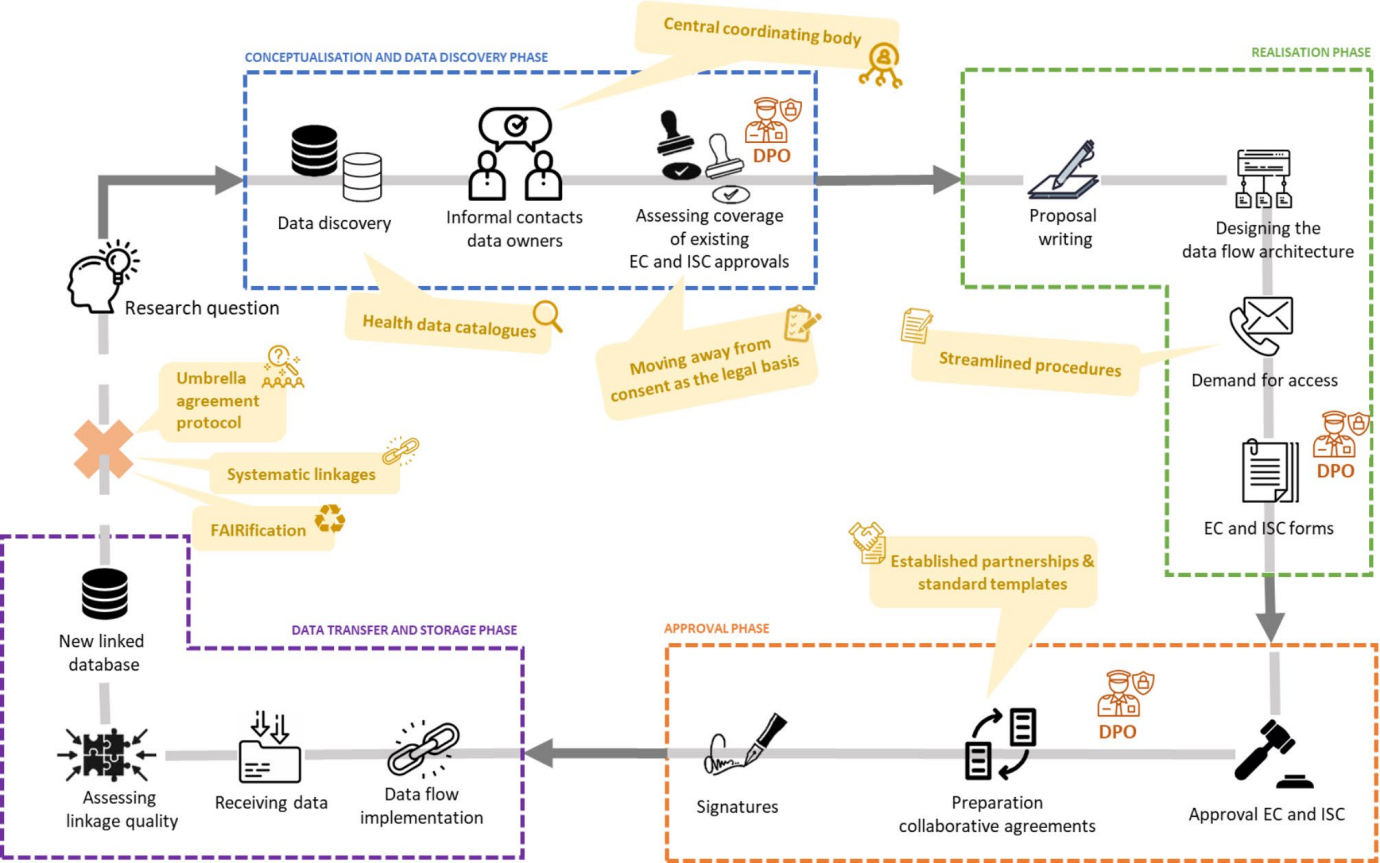
Recommendations to facilitate the consecutive steps of a data user’s journey to implement a data linkage study. ICF: Informed Consent Form; TTP: Trusted Third Party; SPE: Secure Processing Environment; DPO: Data Protection Officer; EC: Ethics Committee; ISC: Information Security Committee; FAIR: Findable, Accessible, Interoperable and Reusable

Besides these recommendations for researchers working within the current environment, broader systemic changes could further enhance processes. These are briefly summarised in Fig. 6. Each phase of the process, ranging from conceptualisation and data discovery to securing approvals and finalising agreements, offers opportunities for improving efficiency, enhancing stakeholder collaboration, and addressing legal and ethical challenges. By examining these aspects both at the national and EU level, valuable insights can be gained for streamlining workflows and better navigating complex regulatory environments in future data linkage studies.

Emerging European frameworks and infrastructures are being developed to help addressing these existing barriers by harmonising regulations and promoting interoperability. In the field of genomics, the ‘1 + Million Genomes (1 + MG)’ initiative aims to enable secure access to genomics and corresponding clinical data across Europe. To support this initiative, the Genomic Data Infrastructure (GDI) project has been launched to establish a federated data infrastructure in Europe, however, at present, an operational system ensuring secure and efficient genomic data access does not yet exist. Its expected alignment with the EHDS regulation will further enhance public health genomics by enabling the secondary use of health data for research through a unified, secure framework for data access and sharing across Europe.

### Establishing comprehensive health data catalogues

The first step for a researcher with a specific research question is to identify relevant data sources and determine how to access them (Fig. 6 – blue box). However, navigating diverse data sources, each managed by different entities, can be challenging and understanding its content and accessibility requires field-specific expertise and knowledge of legal frameworks. Therefore, the availability of metadata and codebooks from primary data sources plays a crucial role in ensuring optimal reuse and minimising redundancy. Further, respecting the principle of proportionality requires a thorough understanding of these data sources upfront, necessitating clear codebooks and the involvement of experts familiar with the data during the variable selection phase. Additionally, researchers must possess an in-depth knowledge of the linked data sources during statistical analysis and interpretation to accurately assess their limitations.

The Belgian Health Data Agency (HDA) was established by law the 14th of March 2023 and is reshaping the Belgian health data landscape by facilitating the secondary use of electronic health data, thereby supporting the implementation of the EHDS. To address the challenges of data discovery, the HDA is introducing a comprehensive data catalogue, a centralised repository that stores metadata and information about health-related data assets in Belgium. It will enable users to discover, understand, and potentially access relevant datasets while ensuring adherence to the FAIR principles (Findable, Accessible, Interoperable, and Reusable).

To further improve its functionality in a cross-border context, the Belgian Health Data Metadata Catalogue is being expanded to support upstream interoperability with the EHDS. By mapping metadata to HealthDCAT-AP standards, it ensures alignment and technical interoperability with the data catalogue in the HealthData@EU Central Platform. This standardisation of metadata not only enhances the discoverability of health data but also enables seamless cross-portal searches. Developing consensus on metadata standards for the core description of genomic datasets remains an ongoing topic of discussion.

### Installing a central coordinating body

To navigate the data landscape and identify the required administrative procedures, it was necessary to search for contact points and rely on informal networks to gather information. This approach is not only inefficient, the reliance on personal or ad hoc connections lacks transparency and can create barriers for those without established networks, leading to unequal access to data. While personal connections and collaborations are often necessary as they provide valuable insights, a more structured and centralised coordination system could be helpful to coordinate the communication between different stakeholders and streamline administrative workflows.

The Belgian HDA will act as a central coordination body and will offer services that could address these issues, such as supporting the communication between data holders and data users, and guiding data users in preparing the request for access to health data. It can also assist data users in analysing legal compliance for accessing healthcare data for the purposes intended by the data user. This is in line with the EHDS regulation requiring each member state to establish one or more Health Data Access Bodies (HDABs) to facilitate data availability for scientific research, innovation, and policy. Acting as intermediaries, the HDABs will help researchers connect with relevant data providers and facilitate knowledge exchange, simplifying the data access process. However, individual collaborations will still be needed to a certain extent to fully understand specific research needs and effectively interpret the mobilised data.

One key challenge that the EHDS regulation does not fully address is cross-dataset linkage across different data sources. While HDABs will streamline access to individual datasets, the actual linkage of data from multiple providers remains complex, especially when dealing with different governance frameworks. Additional mechanisms and a stable infrastructure are needed to enable secure, interoperable, and privacy-preserving linkages across multiple data sources.

### Moving away from consent as the legal basis

Once the relevant data sources have been identified, assessing the coverage of existing ethical and legal frameworks is required. Many of the legal barriers inhibiting data linkages are due to a lack of clarity and differing interpretations. For example, when is it necessary to renew informed consent? Under which circumstances is data linkage considered to be in the public interest? How do we balance data protection considerations with sufficient data utility for legitimate purposes? Traditionally, different types of consent have been proposed as an appropriate tool to respect the rights of natural persons and an effective legal ground for data processing and linkage. Broad consent was explored as an option after the implementation of the GDPR, but recently rejected as a legal ground and questioned as an ethically useful tool [15]. Dynamic consent offers a lot of promise as a legal ground because it can be very precise, but it is burdensome for both researchers and participants and may undermine the ethical significance of consent due to consent fatigue leading to a high attrition rate and a bias in the available data [16]. An alternative approach to consent is the opt-out model, commonly used for disease registries, where individuals are automatically included unless they actively choose to opt-out. However, this model assumes that individuals are informed about the secondary uses and are aware of their rights to withdraw.

Within the EHDS, the current preference is to build an opt-out model of consent to access health data for secondary use, which offers some ethical advantages if citizens are sufficiently aware and informed, but is not precise enough to be used as a legal ground [17]. For genomic data, an extended transition period is foreseen, with secondary use provisions for human genetic data only applying from 2031. Member states may also impose stricter measures and additional safeguards, reflecting the sensitivity of genomic information.

From an ethical perspective, the solution may be to appreciate informed consent as a continuum and move from a fully to an appropriately informed consent model in genomic research [18]. From a legal perspective, moving away from consent as a legal basis may pave the way to more structural solutions for secondary use and linkage of health data in the public interest [9]. This would allow for a shift from control and consent based practices to a framework built on trust. Under the Data Governance Act, individuals can – supported by data altruism organisations – voluntary donate their data for public interest purposes through the data altruism framework. This approach could reduce the need for case-by-case consent, by introducing a broad, standardised consent framework that allows for pre-approved, continuous, and responsible data (re-)use for the societal benefit.

Building and maintaining citizens’ trust in the responsible secondary use of their data requires a commitment to operating in line with core patient values. Ideally, incorporating provisions for potential future data linkages in the primary informed consent forms, leveraging patient approved mechanism for data linkage, and fostering structural citizen engagement can further enhance trust and ensure ethical compliance [19]. Moreover, organisations should regularly re-evaluate and update their practices to adapt to evolving privacy concerns and technological advancements. The fast-evolving field of data intensive health research requires to make proper use of flexibilities within the law that allow data linkage and encourage the enforcement of oversight bodies that can accommodate and interpret that flexibility [20].

### Streamlining administrative and legal procedures

Once the study details are fully established, the next step is the realisation phase (Fig. 6 – green box), which involves preparing the necessary documents and forms. The variability of procedures across institutions, even in the same country and sector, complicates data access requests. Different stakeholders, such as healthcare providers, researchers, and data custodians, often have conflicting views on who should control and access the data. Many data holders, such as official statistical offices and other data sources, lack the resources to efficiently handle data requests, leaving researchers struggling to gain access. Additionally, current data governance frameworks do not always prioritise research use cases, underscoring the need for better alignment between governance practices and research needs. Establishing infrastructures with a robust data governance structure, such as setting up a governance board involving different institutions, is essential to build trust among the various data providers and to ensure that data sharing processes are transparent and equitable.

Cross-border data sharing adds another layer of complexity, necessitating harmonised standards and regulations to ensure data protection across different jurisdictions. In the EU context, these challenges are being addressed through initiatives such as the European Data Governance Act, which aims to facilitate data sharing across sectors and member states. The HealthData@EU Central Platform will introduce common application forms to simplify and standardise data access requests, promoting more efficient and effective data sharing across Europe. Subsequently, the HDABs established at the national levels will serve as a central access point to the EHDS, streamlining the process for data requests and utilisation while maintaining a decentralised governance for data access decisions. In the framework of GDI, the creation of a new legal structure known as the European Digital Infrastructure Consortium (EDIC) will aid member states by providing a legal framework that simplifies agreements and establishes common standards for cross-border genomic data sharing.

### Standardising collaborative agreements

After all relevant applications have been prepared and submitted, the study moves into the approval phase (Fig. 6 – orange box). Setting up collaborative agreements, such as confidentiality contracts and DTAs, is often a time-consuming and complex process, particularly because researchers typically do not have the necessary legal expertise to navigate these requirements efficiently. Institutions and their legal departments often insist on the use of their own templates, sometimes on a case-by-case basis. Negotiating these agreements can involve extensive back-and-forth discussions with legal departments, further delaying research timelines.

To streamline this process, the use of standardised templates and having pre-established partnerships or agreements in place can significantly reduce the administrative burden. Many projects already highlighted the benefits of standardising contracts, but achieving harmonisation remains challenging due to variations in data sharing contexts, including different data types, actors, and partnerships. A practical approach is to have a list of clauses commonly needed in these agreements to satisfy GDPR compliance at hand, while allowing for some project-specific information to maintain flexibility [21].

### Preparing for data transfer and storage

After obtaining legal and ethical clearance and securing agreements between all partners, data exchange can proceed (Fig. 6 – purple box). Sufficient storage capacity and computing power must be ensured when dealing with genomic data, while also enabling secure access through robust security and data protection measures. Secure Processing Environments (SPEs) offer a controlled setting for safe data analysis, ensuring that sensitive information is protected while allowing researchers to conduct their work efficiently. Additionally, it is important to decide which file types to retain for optimal data management. For example, ONT POD5 files may be retained to allow for rebasecalling, providing flexibility for future data reprocessing and ensuring high-quality analysis. Alternatively, the MPEG-G standard can be adopted for its ability to compress genomic data more efficiently. This format reduces storage costs and facilitates faster data transmission, which is especially beneficial in a collaborative research environment where data is frequently shared and accessed across multiple institutions. The federated European GDI will comprise a set of national nodes each of which hosts a subset of the data available within the 1 + MG infrastructure.

### Ensuring the long-term sustainability of data linkages

To ensure the long-term effectiveness and efficiency of studies on linked data, it is essential to focus on its sustainability. The extensive and time-consuming procedures required to implement data linkages significantly impact the timeliness of research, making it challenging to address continuously evolving, policy-relevant questions promptly. Obtaining approvals from ethical and legal committees involves lengthy and cumbersome processes. Additionally, the TTP linkage requires signing agreements between all parties, adding further delays. For each new research question, a separate approval from the ISC and EC is often needed, which further prolongs the process. To address this, establishing an “umbrella” agreement protocol for public institutions like Sciensano could streamline research approvals. Such a protocol would cover a broad range of related studies, minimising the need for multiple ad hoc approval processes. Even more effective would be the creation of a national law that regulates data access and linkage for public health genomics research at Sciensano, facilitating more efficient and consistent processes. Moving away from ad hoc linkages towards systematic linkages would also improve the sustainability of linked datasets, avoiding the inefficient “link and destroy” model.

In order to transition from project-based linkages towards a more systemic approach, a stable infrastructure is essential to facilitate the linkage and access to a broad portfolio of data sources relevant to public health genomics research. This requires establishing a resource of representative human genome sequences and integrating it with population-based datasets including health data, social welfare data, socio-economic data and environmental data. In addition, adopting a longitudinal design and foreseeing opportunities for additional sampling and new data collections, as envisioned in the BELCOHORT project (www.belcohort.be), could create a large population cohort in Belgium that can be enriched with individual-level linkages and new data collections over time.

Legal and regulatory frameworks of data (re)use are continuously evolving, reflecting advancements in technology, shifts in public expectations, and policy changes. Navigating these frameworks is a complex task for researchers and organisations, as they must ensure that their data practices remain compliant with current regulations. Facilitating broad data use while safeguarding individual rights highlights the need for a nuanced understanding of the legal landscape. It also underscores the importance of developing robust data governance strategies that can flexibly adapt to new regulations.

## Conclusions

Linking genomic data with existing population-based observational datasets offers significant opportunities for public health genomics but remains a complex and time-consuming process. A Belgian pilot study assessed the feasibility of such linkages, offering recommendations for each phase of the data user journey, from data discovery to securing approvals and agreements. The study highlights opportunities to improve efficiency, stakeholder collaboration, and address legal and ethical challenges. Key recommendations include establishing interoperable data catalogues to facilitate data discovery, creating a central body to increase transparency and coordinate stakeholder communication, standardising data access requests to simplify legal procedures, forming pre-established collaborative agreements to reduce administrative burdens, and planning for sustainability beforehand to move towards structural secondary use of health data. While some findings are specific to the Belgian context, the challenges encountered are not unique to Belgium; researchers worldwide can face similar complexities in their own jurisdictions. Starting with smaller-scale linkages to demonstrate feasibility before scaling up provides a foundation to refine processes and inform more sustainable implementation. Therefore, the insights from this Belgian pilot study are relevant on a broader scale and offer practical guidance for researchers planning integrated public health genomics studies.

## Electronic supplementary material

Below is the link to the electronic supplementary material.

Supplementary Material 1: Pilot study preparation timeline. A table including all consecutive steps and respective dates of the pilot study’s preparation, ranging from concept to approval. (DOCX 52KB)

## Data Availability

No datasets were generated or analysed during the current study.

## Abbreviations

1 + MG: 1 + Million Genomes
B1MG: Beyond 1 Million Genomes
BELHES: Belgian Health Examination Survey
BELHIS: Belgian Health Interview Survey
BCR: Belgian Cancer Registry
DMP: Data Management Plan
DPA: Data Protection Authority
DPIA: Data Protection Impact Assessment
DPO: Data Protection Officer
DTA: Data Transfer Agreement
EC: Ethics Committee
EDIC: European Digital Infrastructure Consortium
EHDS: European Health Data Space
FPS: Federal Public Service
GDI: Genomic Data Infrastructure
GDRP: General Data Protection Regulation
HDA: Health Data Agency
HDAB: Health Data Access Body
ICF: Informed Consent Form
INDELS: Insertions and Deletions
IMA: Intermutualistic Agency
INAMI: National Institute of Health and Disability Insurance
ISC: Information Security Committee
MTA: Material Transfer Agreement
NRN: National Register Number
PC: Project Code
PRS: Polygenic Risk Scores
RNPP: National Register of Natural Persons
RWD: Real-World Data
SCRA: Small Cell Risk Analysis
SNP: Single Nucleotide Polymorphism
SPE: Secure Processing Environment
TTP: Trusted Third Party
VIKZ: Vlaams Instituut voor Kwaliteit van Zorg
WGS: Whole-Genome Sequencing
