# Supplementary material for: From concept to approval: human genomic data integration with population observational data – insights from a Belgian pilot study
Source: Arch Public Health. 2025 Apr 24;83:112. doi: 10.1186/s13690-025-01581-9 (PMC12020153; doi:10.1186/s13690-025-01581-9)
Supplement: Supplementary file 1 — Supplementary Material 1: Pilot study preparation timeline. A table including all consecutive steps and respective dates of the pilot study’s preparation, ranging from concept to approval. (DOCX 52KB) [file 13690_2025_1581_MOESM1_ESM.docx]

**Additional file 1**. Timeline of all consecutive steps of the pilot study's preparation, ranging from concept to approval.

| **Date** | **Phase** | **Action** |
| --- | --- | --- |
| January 2023 | Conceptualisation & data discovery | Contacting **EC Ghent (HIRUZ)** to ask clarification on the coverage of the existing BELHES EC approval and ICF |
|  |  | First meeting with **Sciensano’s DPO** to explain the planned study and ask for advice |
|  |  | Contacting **Sciensano’s legal office** to ask advice regarding the recontacting of study participants |
|  | Realisation | Start drafting first version of the **study protocol** |
| February 2023 | Conceptualisation & data discovery  + Realisation | Selecting variables from the **BELHIS and BELHES 2018 database** by consulting the codebook, and submitting a data request demand (with justification) to the HIS team |
| March 2023 | Realisation | First version of the **Data Management Plan** (DMPOnline) |
|  |  | *March 9^th^ 2023: First submission to the* ***EC Ghent*** |
| April 2023 | Conceptualisation & data discovery | First meeting with **BCR** to discuss a potential data linkage study involving cancer registry data |
|  |  | First contact with **Statbel** to introduce a potential data linkage study involving Statbel data |
|  |  | First meeting with **eHealth** to discuss the data flow for a potential data linkage study |
|  | Realisation | *April 5^th^ 20243: Feedback from* ***EC*** *Ghent (HIRUZ)* |
| May 2023 | Conceptualisation & data discovery | Meeting with EC Ghent (HIRUZ) to understand the best way forward (decision on the scope of the demand, e.g. prospective vs. retrospective data collection) |
|  | Realisation | Changed scope of the study (including data linkages with BCR and Statbel) and start drafting the second version of the **study protocol** |
|  |  | Start drafting the **ISC** demand |
| June – July 2023 | Conceptualisation & data discovery | Receiving feedback from the **BCR** on the study protocol and ISC demand |
|  |  | Second meeting with the **BCR** to discuss the study protocol, BCR codebook, ISC demand and further steps |
| August 2023 | Realisation | Sending **ISC** demand to **Sciensano’s DPO** |
| October 2023 | Conceptualisation & data discovery | Receiving feedback from the **BCR** on the study protocol and ISC demand |
|  |  | Second meeting with **eHealth** to discuss the data flow scheme |
|  |  | Receiving feedback from **Statbel** on the ISC demand |
|  |  | Contacts with the statistician of **Statbel** to finalize the list of selected variables |
|  |  | Receiving feedback from **Sciensano’s DPO** on the ISC demand and meeting to discuss next steps |
|  | Realisation | Finalising **data flow scheme** |
|  |  | Contacting IMA for the **SCRA** |
|  |  | Creation of the **Processing Activity Assessment** template by **Sciensano’s DPO** |
|  |  | Creation of the **DPIA** template by **Sciensano’s DPO** |
| November 2023 | Realisation | Submission of SCRA demand to the “SCRA pool” by **IMA** |
|  |  | Confirmation that the SCRA will be conducted by **VIKZ** |
|  |  | Final version of the **Data Management Plan** (DMPonline) |
|  |  | Final version of the **Processing Activity Assessment** |
|  |  | Start drafting the Microdata request form of **Statbel** |
| December 2023 | Realisation | *4^th^ December 2023: submission to EC Ghent* |
|  |  | Final version of the **DPIA** |
|  |  | Final version of the Microdata request form of **Statbel** (reviewed by DPO) |
| January 2024 | Realisation | Sending the Microdata request form and ISC demand to **Statbel** for informal approval |
|  |  | Sending the Microdata request form to **Statbel**’s DPO committee |
|  | Approval | 15^th^ January 2024: Receiving approval from **Statbel**’s DPO committee |
|  |  | **Statbel** sends confidentiality contract to Sciensano |
|  |  | Informal approval for SCRA by **VIKZ** |
|  | Realisation | *January 30^th^ 2024: Submission to the* ***ISC*** *by Sciensano’s DPO* |
| February – March 2024 | Realisation | *February 13^th^ 2024: Interview with the EC Ghent Review Committee* |
|  |  | February 15^th^ 2024: Additional questions from the **EC** Ghent – Reply on February 29^th^ 2024 |
|  |  | February 28^th^ 2024: Email with additional questions from the **ISC** – Reply the same day |
|  |  | March 15^th^ 2024: Email with additional questions from the **ISC** – Reply on March 18^th^ 2024 |
|  | Approval | *March 19^th^ 2024: Final approval by the* ***EC*** |
| May – June 2024 | Approval | *May 3^rd^ 2024: Final approval by the* ***ISC*** |
|  |  | May 22^nd^ 2024: Sending draft DTA to the **BCR** |
|  |  | June 28^th^ 2024: Confidentiality agreement with **Statbel** signed |
|  |  | June 28^th^ 2024: Receiving revised DTA from **BCR** |
| August 2024 | Approval | August 29^th^ 2024: Signed DTA with **BCR** |
| September – January 2025 | Approval | September 24^th^ 2024: Draft **TTP** Global Document received from eHealth |
|  |  | January 11^th^ 2025: Signed **TTP** Global Document |
